# Nicotine, aerosol particles, carbonyls and volatile organic compounds in tobacco- and menthol-flavored e-cigarettes

**DOI:** 10.1186/s12940-017-0249-x

**Published:** 2017-04-27

**Authors:** Mi-Sun Lee, Ryan F. LeBouf, Youn-Suk Son, Petros Koutrakis, David C. Christiani

**Affiliations:** 1 Environmental and Occupational Medicine and Epidemiology Program, Department of Environmental Health, Harvard T. H. Chan School of Public Health, 665 Huntington Ave, Building I Room 1401, Boston, MA 02115 USA; 2 Centers for Disease Control and Prevention (CDC), National Institute for Occupational Safety and Health (NIOSH), Respiratory Health Division, Field Studies Branch, Morgantown, WV USA; 3 Research Division for Industry & Environment, Advanced Radiation Technology Institute, Korea Atomic Energy Research Institute, Daejeon, South Korea; 4 Exposure, Epidemiology and Risk Program, Department of Environmental Health, Harvard T. H. Chan School of Public Health, Boston, MA USA; 5 Massachusetts General Hospital/Harvard Medical School, Boston, MA USA

**Keywords:** Nicotine, Particles, Carbonyls, VOCs in e-cigarette emissions

## Abstract

**Background:**

We aimed to assess the content of electronic cigarette (EC) emissions for five groups of potentially toxic compounds that are known to be present in tobacco smoke: nicotine, particles, carbonyls, volatile organic compounds (VOCs), and trace elements by flavor and puffing time.

**Methods:**

We used ECs containing a common nicotine strength (1.8%) and the most popular flavors, tobacco and menthol. An automatic multiple smoking machine was used to generate EC aerosols under controlled conditions. Using a dilution chamber, we targeted nicotine concentrations similar to that of exposure in a general indoor environment. The selected toxic compounds were extracted from EC aerosols into a solid or liquid phase and analyzed with chromatographic and spectroscopic methods.

**Results:**

We found that EC aerosols contained toxic compounds including nicotine, fine and nanoparticles, carbonyls, and some toxic VOCs such as benzene and toluene. Higher mass and number concentrations of aerosol particles were generated from tobacco-flavored ECs than from menthol-flavored ECs.

**Conclusion:**

We found that diluted machine-generated EC aerosols contain some pollutants. These findings are limited by the small number of ECs tested and the conditions of testing. More comprehensive research on EC exposure extending to more brands and flavor compounds is warranted.

## Background

Electronic cigarettes (referred to as ‘ECs’ hereafter) deliver nicotine with flavorings and other additives via inhalation without combustion. They are marketed as an alternative to conventional cigarettes [1]. In August 2016, a new U.S. Food and Drug Administration (FDA) regulation was promulgated to regulate all tobacco products including ECs, and the sale of these products was banned to people under age 18 years (http://www.fda.gov/TobaccoProducts/Labeling/RulesRegulationsGuidance/ucm394909.htm).

Although the FDA has not yet developed standards for testing or for acceptable emissions, current evidence on the emission from EC smoking raises health concerns. Wide ranges in the levels of chemical substances, such as nicotine, tobacco-specific nitrosamines, aldehydes, metals, volatile organic compounds (VOCs), phenolic compounds, polycyclic aromatic hydrocarbons, flavors, aerosol particles, and solvent carriers have been reported in various EC matrices, including refill solutions, cartridges, aerosols and environmental emissions (reviewed in [2, 3]). Particle number concentration was found to be similar or higher in EC emissions than in conventional tobacco cigarette smoke [4]. VOCs (e.g., benzene classified as a known human carcinogen for all routes of exposure by EPA) and carbonyl compounds (e.g., formaldehyde, acetaldehyde, and acrolein) were at lower concentrations in EC emissions than in conventional cigarettes [5–7]. Heavy metal (e.g., lead and nickel) concentrations in EC emissions were equal to or higher than concentrations in conventional cigarettes [8].

However, current evidence on emission profiles is of limited use for risk assessment in the US population. Many studies have analyzed chemicals in refill solutions and cartridges rather than in EC emissions and can only infer possible emission levels [2]. Cheng [2] has stated that there is a “strong need for evaluation of products currently on the US market”, indicating that there is little information available for US products, especially regarding their flavor-based emission profiles.

Therefore, our study aimed to assess the content of emissions for ECs with flavors (tobacco and menthol) that dominated the U.S. market (95%>) in 2013 [9]. We considered whether flavor and puffing time affect the emission of five groups of chemical compounds present in tobacco smoke: nicotine, fine particle fractions, including nanoparticles or ultrafine particles (PM_0.1_ or UFPs, particulate matter of aerodynamic diameter less than 100 nm) and fine particles (PM_2.5_, particulate matter of aerodynamic diameter less than 2.5 μm), particle number concentration (PNC, particle size range from 0.02 to 1 μm), carbonyls, VOCs, and trace elements.

## Methods

### EC aerosol generation system

**Fig. 1:**
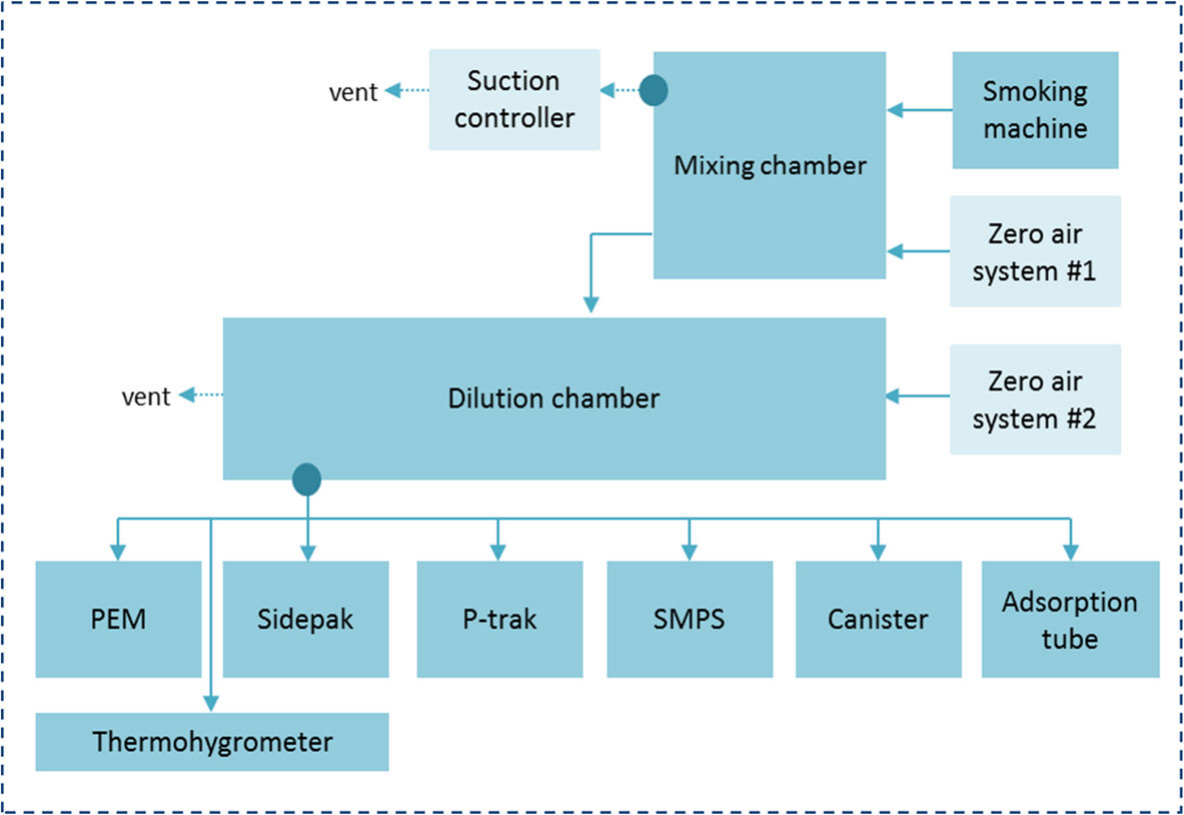
EC aerosol generation and sampling system

EC aerosol generation and sampling was performed in the Environmental Chemistry Laboratory at the Harvard T.H. Chan School of Public Health where thermo-hygrometric conditions were continuously monitored. We used two dominant flavors, tobacco and menthol, of the EC V2 brand (VMR Products, LLC), a popular U.S. brand, containing a nicotine strength of 1.8%, which is a popular “strength” consumed by experienced EC users [10] and is close to the maximum level set by the European Commission regulatory proposal [11] (http://www.europarl.europa.eu/pdfs/news/expert/infopress/20131216IPR31001/20131216IPR31001_en.pdf). The EC devices used in this study were rechargeable “cigalike” devices consisting of a cartomizer, which combines a disposable cartridge (which holds a liquid solution) and a built-in atomizer, and a rechargeable battery with a glowing red light-emitting diode (LED) tip on the end that lights up with each puff and serves as an indicator of use. This device has the same components as many EC devices. The tip of the cartridge served as the EC’s mouthpiece. The liquid in the cartridge was heated by a battery and turned into a vapor by an atomizer. The EC aerosol generation system consisted of a smoking machine, mixing chamber, dilution chamber, suction controller and two zero air systems, as shown in Fig. 1. A dilution chamber was used to generate stable concentrations of machine-generated aerosols with nicotine levels similar to those in a general indoor environment. As targeted, the concentrations of nicotine generated from our smoking machine were within the range of previous studies [12–15]. The automatic multiple smoking machine (Modified TE-2 system, Teaque Enterprises, CA, USA) was used to generate EC aerosols. This is a multiple port linear piston-like smoking machine with adjustable and very wide-ranging puffing regimes. The determination of the characteristics of machine-generated EC emissions was done under controlled conditions of temperature, relative humidity and air exchange rate [16]. The EC aerosol generated from the smoking machine was entered into a mixing chamber at a flow rate of 1.3 L/min and mixed with pure air through zero air system #1 (10 L/min). The volume and air exchange rate of the chamber was 442.5 L (0.762 × 0.762 × 0.762 m) and 1.53 h^−1^, respectively. The mixed EC aerosol at 4.3 L/min was entered into a dilution chamber to adjust for concentrations of airborne markers, such as PM_2.5_. At this time, additional pure air (81 L/min) generated by zero air system #2 was supplied to the dilution chamber to reduce the EC-emitted aerosol concentration. Consequently, the total dilution ratio of the system was 1:172 calculated by multiplying the dilution factors of the mixing (8.69) and dilution (19.8) chambers. Experiments on mixing patterns and equilibrium times of EC aerosols in the system were carried out, and it was found that EC aerosol levels in the system were consistent after 40 min of smoking machine operation to allow for equilibrium. To improve reliability of the experimental results, the system was purged with pure air. The EC aerosol was collected and analyzed using various instruments to evaluate the effect of flavor and puffing time on the aerosol characteristics. During the experimental period, the relative humidity and temperature inside the chamber were controlled at 18.8 ± 6.7% and 34.4 ± 0.9 °C, respectively. All experiments were carried out using a smoking machine with two ECs at different puff rate(s), 1 puff and 2 puffs per min, and each experiment was repeated.

### Analysis of EC aerosols

#### Nicotine

Nicotine emitted from ECs was collected by XAD-7 sorption tubes (SKC, Inc., Eighty Four, PA, USA) at a flow rate of 1 L/min for 60 min. The tubes were desorbed completely using 2.0 mL of 2-propanol. The nicotine concentrations were measured by a 7890A gas chromatograph (Agilent Technologies, Santa Clara, CA, USA) equipped with a 5975C mass selective detector (Enthalpy Analytical, Inc., NC, USA). A Second Source sample (Nicotine SS) was analyzed along with the samples as a Laboratory Control Sample (LCS). The recovery was 108%.

*PM*_*2.5*_ *.* The real-time mass concentration of PM_2.5_ was measured using the SidePak^TM^ light-scattering integrating nephelometer (Model AM510, TSI, Inc., MN, USA). The one-hour (h) limit of detection (LOD) of the SidePak is estimated to be 3 μg/m^3^. Since the SidePak generally overestimates PM_2.5_ concentrations [17, 18], SidePak measurements were calibrated using integrated PM_2.5_ concentrations obtained by using a co-located Personal Exposure Monitor (PEM), which is a small inertial impactor designed specifically for personal monitoring [19]. The flow rates of all PEMs were 9 L/min. PEMs collected PM_2.5_ on 37-mm Teflon filters placed downstream of a size-selective inlet that uses a greased impaction plate. Teflon filters were weighed on an electronic microbalance (Cahn Model C-31, Cahn Instruments, Madison, WI) in duplicate before and after sample collection. Filters were equilibrated in a controlled temperature (71 ± 3 °F) and relative humidity (40 ± 5%) room, both before and after sampling. To eliminate the effects of static charge, the Teflon filters were passed over Po_210_ sources (alpha rays) prior to each weighing. The integrated PEM PM_2.5_ mass concentration is the net mass collected on the filter (μg) divided by the sample volume of air (m^3^), based on measured flow and duration.

We calculated the calibration factor (CF) using the average value measured by SidePak divided by the PEM concentration (Eq. 1):

1 $$ C F = \frac{SidePa{ k}_{average}}{Integrated\  PEM\  Concentration} $$

To determine the calibration-adjusted SidePak value for each 10-s measurement, we divided the measured value by the CF.

#### Particle number concentration (PNC) and Nanoparticles

The PNC was analyzed using the P-Trak® Ultrafine Particle Counter (TSI model 8525, TSI Inc., Shoreview, USA). The sampling flow rate and interval of P-Trak were 0.1 L/min and 10 s, respectively. Nanoparticle masses and number concentrations were measured over consecutive 5-min internals using the TSI Model 3936 Scanning Mobility Particle Sizer (SMPS) system consisting of the TSI Electrostatic Classifier (Model 3080) and TSI Long Differential Mobility Analyzer (Model 3081) equipped with a water-based Condensation Particle Counter (CPC, Model 3785). The sampling flow rate of the SMPS was 0.3 L/min and the range of measurement was 10 to 1000 nm. We assumed a particle density of 1.2 g/cc.

#### Volatile organic compounds (VOCs) and Carbonyls

VOCs and carbonyls were analyzed using a draft National Institute for Occupational Safety and Health (NIOSH) canister method [20] that detects the following compounds: acetaldehyde, acetone, ethanol, acetonitrile, isopropyl alcohol, benzene, toluene, methylene chloride, 2,3-butanedione, n-hexane, chloroform, 2,3-pentanedione, methyl methacrylate, 2,3-hexanedione, ethylbenzene, m,p-xylene, styrene, o-xylene, alpha-pinene, and d-limonene. Potentially toxic carbonyls such as acetaldehyde can form when e-liquids are heated to high temperature [5]. Acetaldehyde is classified as possibly carcinogenic to humans (Group 2B) by the International Agency for Research on Cancer (IARC) [21] and is the most abundant carcinogen in tobacco smoke. Benzene is classified as carcinogenic to humans (Group 1) by IARC [22]. Fused-silica lined canisters (6 L, Entech Instruments, Simi Valley, CA) were used to collect VOCs for 60 min. Canisters were shipped to the NIOSH-Morgantown Organics Laboratory for analysis after pressurization with UHP nitrogen. A canister autosampler/preconcentrator (7016D/7200, Entech Instruments) coupled with a GCMS system (6890/5975, Agilent Technologies) was used to concentrate a 250 mL sample. Internal standards, bromochloromethane, 1,4-difluorobenzene, and chlorobenzene-d5, were used to quantify target analytes based on response factors.

#### Trace elements

An energy dispersive X-ray fluorescence (EDXRF) spectrometer was used to determine the concentrations of 48 trace elements in a range of atomic numbers from 11 (Na) to 82 (Pb), including Ag, Al, As, Au, Ba, Br, Ca, Cd, Ce, Cl, Co, Cr, Cs, Cu, Eu, Fe, Ga, Ge, Hg, K, La, In, Mg, Mn, Mo, Na, Nb, Ni, P, Pb, Pd, Rb, S, Sb, Sc, Se, Si, Sm, Sn, Sr, Tb, Ti, Tl, V, W, Y, Zn, and Zr. To do this, we analyzed the characteristics of the Teflon filters used during PEM sampling. Elemental analysis was conducted using an Epsilon 5 EDXRF spectrometer (PANalytical, Almelo, The Netherlands) which utilizes secondary excitation from 10 secondary selectable targets [23]. The spectrometer employs a 600 W dual (scandium/tungsten, Sc/W) anode X-ray tube, a 100 kV generator, and a solid-state germanium (Ge) detector. A total of 49 MicroMatter XRF calibration standard polycarbonate films (Micromatter Co., Vancouver, Canada) were used for calibration of 48 elements. We also used the U. S. National Institute of Standards and Technology (NIST) standard reference material (SRM) 2783 for quality control of the analytical procedure.

### Statistical analysis

All data are presented as the mean ± standard deviation (SD) levels of selected compounds in EC emissions. The difference in EC emission levels between groups (e.g., menthol vs. tobacco flavor, 1 puff vs. 2 puffs) were tested using Student’s *t*-test. In addition, linear regressions were applied to estimate percent changes as (10^β^-1) × 100%, where β is the estimated regression coefficient, and the corresponding 95% CIs for the association between aerosol particle concentrations and type of flavor, adjusting for puffing time. All analyses were performed using SAS (version 9.4; SAS Institute Inc., Cary, NC, USA).

## Results

The content of EC emissions for chemical compounds by flavor and puffing time are presented in Table 1.

**Table 1:** Concentrations of EC emission parameters by flavors and puffing time

	Menthol flavored EC						Tobacco flavored EC					
	EC1P^a^			EC2P^b^			EC1P^a^			EC2P^b^		
	Exp1	Exp2	Mean ± SD	Exp1	Exp2	Mean ± SD	Exp1	Exp2	Mean ± SD	Exp1	Exp2	Mean ± SD
Nicotine, μg/m^3^	0.6	0.5	0.55 ± 0.07	1.5	3.3	2.40 ± 1.27	1.1	2.7	1.90 ± 1.13	1.1	7.6	4.35 ± 4.60
Aerosol particles												
PM_2.5_, μg/m^3^	2.7	1.8	2.25 ± 0.64	24.0	343.8	183.90 ± 226.13	3.7	N.A.	–	21.9	21.1	21.5 ± 0.57
Nanoparticle, ng/m^3^	492.7	497.8	495.25 ± 3.61	1,013.4	1,044.6	1,029.0 ± 22.06	1,230.6	1,655.2	1,442.90 ± 300.24	2,443.2	2,442.0	2,442.6 ± 0.85
Nanoparticle, #/cm^3^	5,430.0	5,631.0	5,530.50 ± 142.13	9,294.0	9,498.0	9,396 ± 144.25	10,940.0	12,688.0	11,814 ± 1,236.02	17,722.0	17,857.0	17,789.5 ± 95.46
PNC, #/cm^3^	7,256.0	8,481.0	7,868.50 ± 866.21	11,929.0	13,651.0	12,790 ± 1,212.64	15,242.0	16,835.0	16,038.5 ± 1,126.42	24,035.0	22,429.0	23,232.0 ± 1,135.61
Carbonyls												
Acetaldehyde, ppb	0.4	<LOD	–	<LOD	<LOD	–	<LOD	<LOD	–	<LOD	<LOD	–
Acetone, ppb	0.4	<LOD	–	<LOD	<LOD	–	<LOD	<LOD	–	1.4	5.3	3.35 ± 2.76
VOCs												
Ethanol, ppb	29.9	13.7	21.8 ± 11.46	<LOD	<LOD	–	108.7	35.5	72.1 ± 51.76	17.8	15.7	16.75 ± 1.49
Acetonitrile, ppb	<LOD	<LOD	–	0.5	<LOD	–	<LOD	<LOD	–	0.9	<LOD	–
Isopropyl alcohol, ppb	<LOD	81.3	–	<LOD	26.8	–	10.5	58.8	34.65 ± 34.15	55.8	49.3	52.55 ± 4.60
Benzene, ppb	2.6	N.D.	–	0.7	1.2	0.95 ± 0.35	0.5	<LOD	–	<LOD	6.6	–
Toluene, ppb	N.D.	N.D.	–	N.D.	N.D.	–	N.D.	N.D.	–	1.5	N.D.	–

*N. D* not detected, *N. A* not available, *LOD* limit of detection, *Exp1* first experiment, *Exp2* repeat analysis of Exp1

^a^Experiments using 2 ECs in the smoking machine at a frequency of 1 puff per min

^b^Experiments using 2 ECs in the smoking machine at a frequency of 2 puffs per min

### Nicotine

Nicotine was identified in all tested EC emissions. The mean concentration of nicotine was 3.13 μg/m^3^ (median: 1.90 μg/m^3^) from tobacco-flavored ECs and 1.48 μg/m^3^ (median: 1.05 μg/m^3^) from menthol-flavored ECs. There were no statistically significant differences in the mean concentrations of nicotine based on flavor or puffing times.

### Aerosol particles (PM_2.5_, nanoparticles, and PNC)

**Fig. 2:**
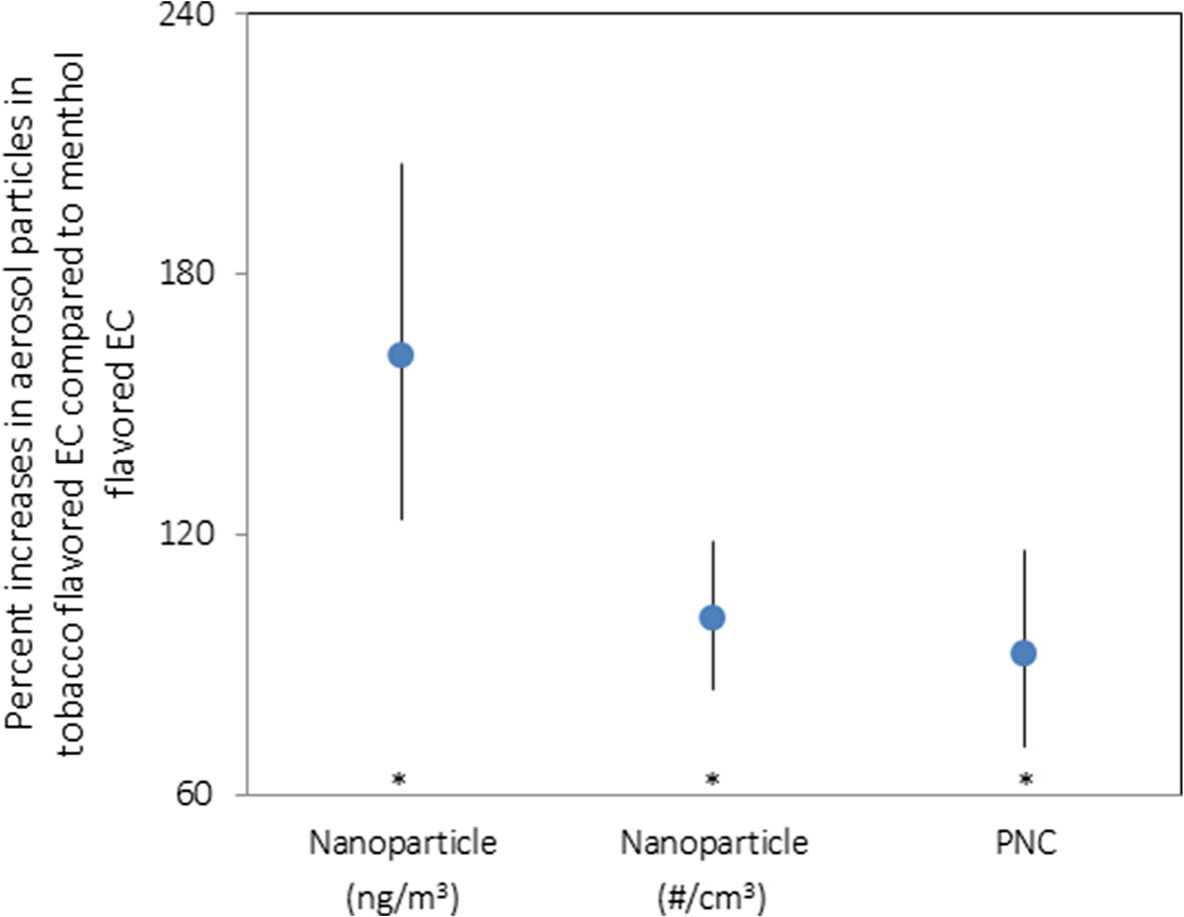
Estimated percent increases and 95% CIs in aerosol particles associated with type of flavor, adjusting for puffing time. *Circle blue symbols* indicate the effect estimate. * *p* < 0.001

Particle mass and number concentrations generated from EC aerosols were identified in all tested ECs. The mean concentration of nanoparticles was significantly higher from tobacco-flavored ECs than from menthol-flavored ECs (1,942.8 vs 762.1 ng/m^3^, *p* = 0.013). For menthol-flavored ECs, a significantly higher mass concentration of nanoparticles was produced by 2 puff/min than by 1 puff/min (1,029.0 vs 495.3 ng/m^3^, *p* < 0.001). There was no significant difference in nanoparticle concentrations based on puffing time for the tobacco-flavored ECs. We found significant differences in the number concentration of nanoparticles based on flavor (menthol: 7,463.2 vs tobacco: 14,801.8 particles/cm^3^, *p* = 0.013) and puffing time. The mean PNC was significantly higher from tobacco-flavored ECs than from menthol-flavored ECs (19,635.3 vs 10,329.3/cm^3^, *p* = 0.012). When stratified by puffing time, 2 puffs/min produced a significantly higher PNC than 1 puff/min for the menthol-flavored ECs (12,790.0 vs 7,868.5 particles/cm^3^, p = 0.024) and for the tobacco-flavored ECs (23,232.0 vs 16,038.5 particles/cm^3^). There was no significant difference in PM_2.5_ concentrations based on flavor or puffing time. When we adjusted for puffing time, tobacco flavoring increased the mass concentration of nanoparticles by 162% (95% CI, 124% to 206%), the number concentration of nanoparticles by 101% (95% CI, 85% to 119%), and the PNC by 93% (95% CI, 72% to 117%) in comparison to menthol flavoring (Fig. 2).

### Carbonyls

The concentration of acetaldehyde was measured from menthol ECs at 1 puff/min, but the other groups had concentrations below the method’s detection limit. The mean concentration of acetone was 3.35 ppb from tobacco ECs at 2 puffs/min, while the other groups had levels mostly below the method’s detection limit.

### VOCs

Of the 18 VOCs, ethanol, acetonitrile, isopropyl alcohol, benzene, and toluene had concentrations above the LOD. Toluene was only detected from tobacco ECs at 2 puffs/min.

### Trace elements

Silicon (Si), Chlorine (Cl), Barium (Ba), and Indium (In) were detected depending on the flavor and puffing time, while other elements were below the method’s detection limit.

## Discussion

Our findings suggest that nicotine, fine and nanoparticles, other toxic chemicals, and carcinogens are present in the emissions from the two dominant flavors of ECs sold in the U.S. In this study, acetaldehyde and benzene were identified at low levels. Tobacco-flavored ECs delivered higher mass and number concentrations of nanoparticles than menthol-flavored ECs.

Our results confirm findings from previous studies in which nicotine, aerosol particles, and other toxic chemicals were detected in EC emission [5, 16]. Nicotine is a highly addictive substance found in cigarette smoke and other tobacco products including ECs. A study by Fu and colleagues in Spain [24] collected 30-min measurements of airborne nicotine as a marker of second-hand smoke (SHS) exposure in various settings including healthcare centers, bars, public administration offices, educational centers, and on public transport. The median concentration of airborne nicotine was 1.36 μg/m^3^ (range: 0.43 to 4.33 μg/m^3^), which is similar to the levels observed in this study (median: 1.30 μg/m^3^, range: 0.50 to 7.60 μg/m^3^). The mean concentration of nicotine generated from ECs in the present study (2.3 μg/m^3^) was somewhat lower than airborne nicotine levels measured for 1 h in indoor offices in the US (mean: 3.8 μg/m^3^) [12], but nicotine levels generated from tobacco-flavored ECs (mean: 3.13 μg/m^3^) in this study were similar. In a study by Baek and colleagues in Korea [13], mean concentrations of airborne nicotine measured for 2 h were 1.8 μg/m^3^ for home indoors, 2.5 μg/m^3^ for offices indoors, and 4.8 μg/m^3^ for restaurants indoors. The nicotine concentrations in EC emissions in the present study were somewhat higher than home indoor concentrations (mean: 1.43 μg/m^3^) [14] and greater than outdoor concentrations measured for 30 min (median: 0.81 μg/m^3^) [15], implying that ECs deliver nicotine in doses that are comparable to secondhand exposure from conventional tobacco cigarettes.

In this study, the median concentration of PM_2.5_ was 21.1 μg/m^3^ (mean: 59.86 μg/m^3^), which is similar to the airborne concentrations in indoor and outdoor assessments of SHS exposure [15, 24] and passive exposure to EC emissions in a simulated café [25]. In this study, there was no significant difference in the concentration of PM_2.5_ based on flavor, but flavor affected PM_2.5_ levels in a prior study [26]. Previous studies reported that ECs deliver high levels of nanoparticles [1, 24, 27, 28], which can penetrate deep into the respiratory tract, reach the alveolar sacs [29, 30] and carry toxic chemicals into the blood stream. These toxic chemicals can then appear in various organs including the liver, kidney, heart and brain [31]. In the present study, nanoparticles were observed in all examined EC emissions. Our study indicates that menthol-flavored ECs produce fewer nanoparticles and lower PNCs than the tobacco-flavored ECs, which is consistent with previously published data [26]. In contrast, a previous study reported that flavors did not change the PNC levels [4]. Based on our results, the tobacco-flavored ECs generated more particles than menthol-flavored ECs. Given a previous report suggesting that particles emitted from ECs have different physical and chemical properties compared to particles in cigarette smoke [32] and that EC aerosols do not contain true particulate matter unlike combustible cigarette smoke [33], fine and ultrafine particles present in EC aerosols are not directly comparable with those in cigarette smoke.

In this study, small amounts of carbonyls and toxic VOCs were detected in EC aerosols. More importantly, carcinogens such as benzene and acetaldehyde were identified. Toluene was detected only in emissions from tobacco-flavored ECs at 2 puffs/min. Acetaldehyde, a major component in the gas phase of tobacco smoke, is of particular interest because of its carcinogenic and genotoxic effects [21]. Acute exposure to acetaldehyde results in irritation of the eyes, skin, and respiratory tract in humans [34]. Acetaldehyde induced DNA and chromosomal damage in human lymphocyte in vitro [35–37]. Our findings are consistent with those from previous studies in which trace amounts of acetaldehyde were detected in EC emissions [1, 5, 16, 25, 38]. Acetaldehyde was found in vapors exhaled in an 8 m^3^ test chamber by volunteer EC users [1]. Geiss and colleagues [16] reported carbonyls were below the method LOD in the air of a30 m^3^ chamberbut were detected when the carbonyls were determined from a gas sampling bag directly connected to the smoking machine. In a study by Goniewicz and colleagues [5], the content of acetaldehyde in emissions from EC cartridges ranged from 0.11 to 1.36 μg per 15 puffs. In this study, acetaldehyde in EC emissions was mostly below the LOD, but was detected at 0.4 ppb from menthol ECs at 1 puff/min. Studies have reported that carbonyl levels generated from the first-generation (cigarette-like) EC devices, as used in our study, were lower than levels found in tobacco cigarette smoke [5, 38]. A recent study has reported that new-generation EC devices at high power produce high levels of aldehyde but only under ‘dry puff’ conditions, which deliver a strong unpleasant taste by overheating the liquid [39].

The presence of VOCs has been reported in EC emissions. Ten of 12 brands emitted detectable levels of toluene and *m,p*-xylene but not benzene [5]. None of these compounds were found in passive exposure to EC emissions [1]. Benzene was detected in EC emissions from volunteer EC users, but it was also found at the same level in background concentrations [25]. In a study by McAuley and colleagues [40], small amounts of benzene, toluene, ethylbenzene, and m,p-xylene were detected above LOD. In this study, five of the analyzed VOCs were detected: ethanol, acetonitrile, isopropyl alcohol, benzene, and toluene. Benzene is a well-known hematotoxic carcinogen that can cause leukemia [22]. While carcinogens are found in trace amount in EC emissions, the effects of EC emission on cancer risk have not been reported.

Here, trace elements such as Si, Cl, Ba, and In were found at trace levels while others were below LOD. Trace amounts of toxic metals such as Cd, Ni, and Pb were present in EC emissions in previous studies, but the same elements were also detected in blank samples [5, 25].

There is a need for caution in interpreting our findings. Exposure limits and standards recommended or set by agencies and organizations such as the Occupational Safety and Health Administration (OSHA), the National Institute for Occupational Safety (NIOSH), the American Conference of Governmental Industrial Hygienists (ACGIH), the U.S. Environmental Protection Agency (EPA), and the World Health Organization (WHO) have been established for the compounds that were detected in EC emissions [41–47] (Table 2). Although airborne levels of carbonyls and VOCs in EC emissions are considerably lower than the occupational standards and guidelines, there are no standards for the general public. The application of occupational exposure limits (OELs) in the workplace to non-occupational exposure in the general public may not be entirely applicable or appropriate for EC users because OELs are set for workers who are at a greater risk than the general population [48]. For PM_2.5_, about half of the samples in our study exceeded the annual mean of NAAQS and WHO guidelines. Thus, our findings should not be interpreted as “safe” or as “acceptable”.

**Table 2:** Exposure limits/guidelines

Agency	Averaging time	PM_2.5_	Nicotine^b^	Acetaldehyde	Acetone	Ethanol	Acetonitrile	Isopropyl alcohol	Benzene	Toluene	Reference
OSHA PEL^†^	8-h	15 mg/m^3,a^	0.5 mg/m^3^	200 ppm (360 mg/m^3^)	1000 ppm (2400 mg/m^3^)	1000 ppm (1900 mg/m^3^)	40 ppm (70 mg/m^3^)	400 ppm (980 mg/m^3^)	1 ppm	200 ppm	OSHA 2016 [41]
OSHA STEL^†^	15-min	NA	NA	NA	NA	NA	NA	NA	5 ppm	NA	OSHA 2016 [41]
NIOSH REL^†^	Up to 10-h	NA	0.5 mg/m^3^	NA	250 ppm (590 mg/m^3^)	1000 ppm (1900 mg/m3)	20 ppm (34 mg/m^3^)	400 ppm (980 mg/m^3^)	0.1 ppm (0.32 mg/m^3^)	100 ppm (375 mg/m^3^)	NIOSH 2016 [42]
NIOSH STEL^†^	15-min	NA	NA	NA	NA	NA	NA	500 ppm (1225 mg/m^3^)	1 ppm	150 ppm	NIOSH 2016 [42]
ACGIH TLV^†^	8-h	10 mg/m^3^	0.5 mg/m^3^	NA	NA	NA	NA	NA	0.5 ppm	NA	ACGIH 2016 [43]
ACGIH STEL^†^	15-min	NA	NA	NA	NA	NA	NA	NA	2.5 ppm	NA	ACGIH 2016 [43]
U.S. EPA NAAQS^‡^	Annual mean	12 μg/m^3^	NA	NA	NA	NA	NA	NA	NA	NA	NAAQS 2012 [44]
	24-h	35 μg/m^3^	NA	NA	NA	NA	NA	NA	NA	NA	NAAQS 2012 [44]
WHO AQG^‡^	Annual mean	10 μg/m^3^	NA	NA	NA	NA	NA	NA	NA	NA	WHO 2005 [45]
	24-h	25 μg/m^3^	NA	NA	NA	NA	NA	NA	NA	NA	WHO 2005 [45]
EU Air Quality Directive^‡^	Annual mean	25 μg/m^3^	NA	NA	NA	NA	NA	NA	5 μg/m^3^	NA	EU 2008 [46]
EU AEI^‡^	3-year mean	20 μg/m^3^	NA	NA	NA	NA	NA	NA	NA	NA	EEA 2015 [47]

*Abbreviations: NA* not available, *PEL* permissible exposure limit, *REL* recommended exposure limit, *TLV* threshold limit value, *STEL* short-term exposure limit, *ppm* parts per million, *OSHA* Occupational Safety and Health Administration, *NIOSH* National Institute for Occupational Safety and Health, *ACGIH*, American Conference of Governmental Industrial Hygienists, *EPA* Environmental Protection Agency, *NAAQS* National Ambient Air Quality Standards, *WHO* World Health Organization, *AQG* Air Quality Guideline, *AEI* Average Exposure Indicator

^†^Occupational exposure limits

^‡^Ambient air quality standards

^a^For particulates not otherwise regulated (total dust)

^b^Noted for skin absorption

It is also important to be cautious about generalizing our results because our findings are limited to the small number of ECs tested and the conditions of testing. Another potential limitation is that the constituents delivered via machine-generated EC emissions may not reflect the emissions exhaled by an EC user, although nicotine levels generated by our smoking machine were found to be similar to those exhaled from EC users [49]. In addition, our results exhibited variation in the content of EC emissions in duplicate experiments. Previous studies have shown large variation in carbonyl concentrations among individual ECs from the same brand [50]. One possible factor to explain our experimental variation is that we used a different cartridge for the duplicate experiments. We suspect that this may contribute to the observed variation in constituent levels and to the range of particle size distribution within products. Alternatively, it is also possible that our simulation system may suffer from different conditioning following the sequence of the simulation. Additional studies based on scientifically validated aerosol generation and chemical analysis methods are needed.

## Conclusions

In summary, EC emissions contain measurable amounts of nicotine, fine and nanoparticles, and other toxic chemicals, implying that EC emissions are a new source of environmental pollution and should be investigated further.

## Abbreviations

ACGIH: American conference of governmental industrial hygienists
CIs: Confidence intervals
EC: Electronic cigarette
EPA: Environmental protection agency
IARC: International agency for research on cancer
LOD: Limit of detection
NIOSH: National Institute for Occupational Safety and Health
OSHA: Occupational Safety and Health Administration
PM_2.5_: Particulate matter of aerodynamic diameter less than 2.5 μm
PNC: Particle number concentration
UFPs: Ultrafine particles, particulate matter of aerodynamic diameter less than 100 nm
VOCs: Volatile organic compounds
WHO: World Health Organization
